# Nutrition in Gynecologic Disease

**DOI:** 10.3390/nu14030707

**Published:** 2022-02-08

**Authors:** Pasquapina Ciarmela

**Affiliations:** Department of Experimental and Clinical Medicine, Università Politecnica delle Marche, 60126 Ancona, Italy; p.ciarmela@univpm.it; Tel.: +39-071-220-6270

The pathologies concerning the gynecological organs are very varied and range from tumoral pathologies to hormonal dysfunctions. The frequency of benign and malignant disease affecting women’s health is very high.

Epidemiological studies show that lifestyle can be an important risk factor for gynecological diseases. One such modifiable lifestyle factor is the diet. The father of medicine, Hippocrates, proclaimed “Let food be the medicine and medicine be the food” almost 25 centuries ago. The relationship between diet and health is yet to be fully explored. The human diet contains a wide variety of plant-based foods that provide essential nutrients for the body. Besides, plant-based foods possess a huge variety of non-nutritive components that offer beneficial health effects.

This book, based on a Special Issue of Nutrients, contains a total of 11 papers (four original research, six reviews and one systematic review) focusing on nutrition in gynecologic disease.

The original articles include studies on uterine and ovarian dysfunctions and placental development.

The first study of this book is in vivo and it focuses on the effect of food supplementation in preventing uterine dysfunction. In detail, it demonstrated that *Spirulina platensis*, an antioxidant blue algae, prevents changes in reactivity and oxidative stress induced by strength training in rat uteri [1]. The second article describe an in vitro study on leiomyoma, the most common benign uterine tumor in reproductive-age women. The commonly used food additive butylated hydroxytoluene (BHT) has a proliferative and fibrotic effect on the ELT-3 leiomyoma cell line. BHT exposure may therefore have a harmful effect on leiomyoma progression and the mechanism may be related to PI_3_K modulation [2].

Following this, an article is included that reports on the possible mechanism that may increase the risk for abnormal placental development in assisted reproductive technologies, studied through tissual evaluation of human placenta and fasting blood nutrient levels from term pregnancy conceived naturally or by intracytoplasmic sperm injection. Hence, in term pregnancy, intracytoplasmic sperm injection impairs placental amino acids transporter expression, cell turnover, and altern umbilical vein levels of specific nutrients [3].

Finally, there is an article on the effect of curcumin on glycemic control and lipid profile in patients with polycystic ovary syndrome (PCOS). Systemic review with meta-analysis and trial sequential analysis suggest that curcumin may improve glycemic control, lipid metabolism, and metabolic abnormality in patients with PCOS [4].

Regarding the reviews, two papers on the effect of phytosteroids (phytoprogestins and phytoestrogens) have been included [5,6]. First, there is a review on possible terapeutic phytoprogestins for female disease studied so far, such as kaempferol, apigenin, luteolin, and naringenin. Although limited data are available, it seems that phytoprogestins could be a promising tool for preventing and treating hormone-dependent diseases [5]. Among the extensively studied phytoestrogens, the second review focuses on genistein, an isoflavone which structurally resembles endogenous estrogen often consumed via soya products [6].

Next, there are two reviews on nutritional aspects in pathological situations, such as functional hypothalamic amenorrhea (FHA) [7], and PCOS [8]. Furthermore, there is a further review on the importance of the nutritional habits for health promotion and lifestyle adaptation to the menopausal period [9].

The last review is a comprehensive general paper that summarizes and furnishes the current perspectives on the importance of nutrition in gynecological disease [10].

The book ends with a systematic review that provides comprehensive and available data on the possible role of phytoestrogens for the treatment of endometriosis [11].

I believe that this collection includes important current studies on the benefits of nutrients in prevention, therapy, and management of female dysfunctions.
